# Comparison of one-year results of photodynamic therapy combined with ranibizumab or aflibercept for treating polypoidal choroidal vasculopathy

**DOI:** 10.1371/journal.pone.0235213

**Published:** 2020-06-24

**Authors:** Arisa Ito, Maiko Maruyama-Inoue, Yoko Kitajima, Shimpei Sato, Tatsuya Inoue, Shin Yamane, Kazuaki Kadonosono

**Affiliations:** 1 Department of Ophthalmology, Yokohama City University Medical Center, Yokohama, Japan; 2 Department of Ophthalmology, Kanto Rosai Hospital, Kawasaki, Japan; Massachusetts Eye & Ear Infirmary, Harvard Medical School, UNITED STATES

## Abstract

**Purpose:**

To compare the 1-year visual outcomes and anatomical responses of patients who received photodynamic therapy (PDT) combined with intravitreal ranibizumab (IVR) injections with those of patients who received PDT combined with intravitreal aflibercept (IVA) injections for treating polypoidal choroidal vasculopathy (PCV).

**Methods:**

We retrospectively studied all treatment-naïve patients with PCV who received PDT combined with either IVR or IVA. Best-corrected visual acuity (BCVA), central macular thickness (CMT), central choroidal thickness (CCT), the number of additional injections, and the presence of polypoidal lesions, as indicated by indocyanine green angiography (ICGA), during 1 year were evaluated.

**Results:**

Forty-four eyes were assessed at the 1-year follow-up examination. Of these, 23 were treated with PDT combined with IVR (PDT/IVR group), and 21 were treated with PDT combined with IVA (PDT/IVA group). In both groups, BCVA was shown to be significantly improved 1 year after the initial treatment. CMT and CCT were also significantly decreased after 1 year. There were no significant differences in the changes in BCVA or CMT between the two groups. However, the change in CCT in the PDT/IVA group was significantly larger than that of the PDT/IVR group (*P* < 0.001). The mean number of additional injections was 0.78 ± 0.21 in the PDT/IVR group and 0.57 ± 0.21 in the PDT/IVA group with no significant difference between the two groups (*P* = 0.45). The polyp regression rate at 12 months was 78.2% in the PDT/IVR group and 78.9% in the PDT/IVA group with no significant difference between the two groups.

**Conclusions:**

PDT combined with either IVR or IVA was well tolerated and appeared to improve both vision and anatomy in patients with PCV. PDT/IVA may have a more pronounced effect on macular choroidal thickness at 1-year follow-up.

## Introduction

Polypoidal choroidal vasculopathy (PCV) is first reported by Yanuzzi et al. [1,2] as a distinct subtype of wet age-related macular degeneration (AMD). PCV is characterized by elevated orange-red lesions with exudation. By indocyanine green angiography (ICGA), hyperfluorescent aneurysmal lesions and a branching choroidal vascular network are typically visible. PCV is also known to be more prevalent in Asians than in Caucasians. [3] In Japan, nearly half of the patients with wet AMD suffer from PCV. [4]

Photodynamic therapy (PDT) with verteporfin is the first sanctioned treatment for neovascular AMD. PDT has a high polyp regression rate in patients with PCV; however, massive hemorrhage and visual deterioration are evident after a prolonged follow-up period. [5–7] Nevertheless, previous studies report that anti-vascular endothelial growth factor (VEGF) agents, such as ranibizumab (Lucentis, Genentech, Inc., South San Francisco, CA, USA) and aflibercept (Eylea, Bayer Health Care, Berlin, Germany) have favorable effects in patients with PCV, although a large number of injections are required to maintain patients’ visual acuity with a lower polyp regression rate than that of PDT. [3,8–14]

Recently, the EVEREST Ⅱ study has reported that combination therapy using ranibizumab and PDT showed more favorable visual outcomes and required fewer injections than ranibizumab monotherapy. [15] Therefore, in recent years, combination therapy for treating PCV has become widespread. Several studies have reported preferable visual outcomes as a result of PDT combined with either intravitreal ranibizumab (IVR) [16–18] or intravitreal aflibercept (IVA). [19–21] However, the anti-VEGF agents optimal for performing combination therapy have not been identified. The purpose of this study is to compare the 1-year visual outcomes and anatomical responses of patients with PCV who received PDT combined with IVR with those of patients who received PDT combined with IVA.

## Materials and methods

We retrospectively analyzed consecutive 44 eyes of 44 Japanese patients aged 50 years or older who were diagnosed with treatment-naïve PCV. All patients were initially treated at Yokohama City University Medical Center between April 2017 and September 2018. This study design was approved by the institutional review board at the Yokohama City University Medical Center. It also followed the tenets of the Declaration of Helsinki. Written informed consent was obtained from all patients prior to use their medical record data in the research.

The inclusion criteria were the presence of PCV determined by clinical findings, spectral-domain optical coherence tomography (SD-OCT) (SPECTRALIS Product Family Version 5.3; Heidelberg Engineering, Germany), and fluorescein angiography (FA) and ICGA (SPECTRALIS Product Family Version 5.3; Heidelberg Engineering, Germany) showing a branching vascular network and polyp-like structures. Patients who had a history of previous treatment for PCV (i.e., laser photocoagulation, PDT, the intravitreal injection of other anti-VEGF agents, or intravitreal steroids) were excluded from this study. Patients who had a history of eye diseases such as uncontrolled glaucoma, macular hole, diabetic retinopathy, and rhegmatogenous retinal detachment, were also excluded.

**Fig 1:**
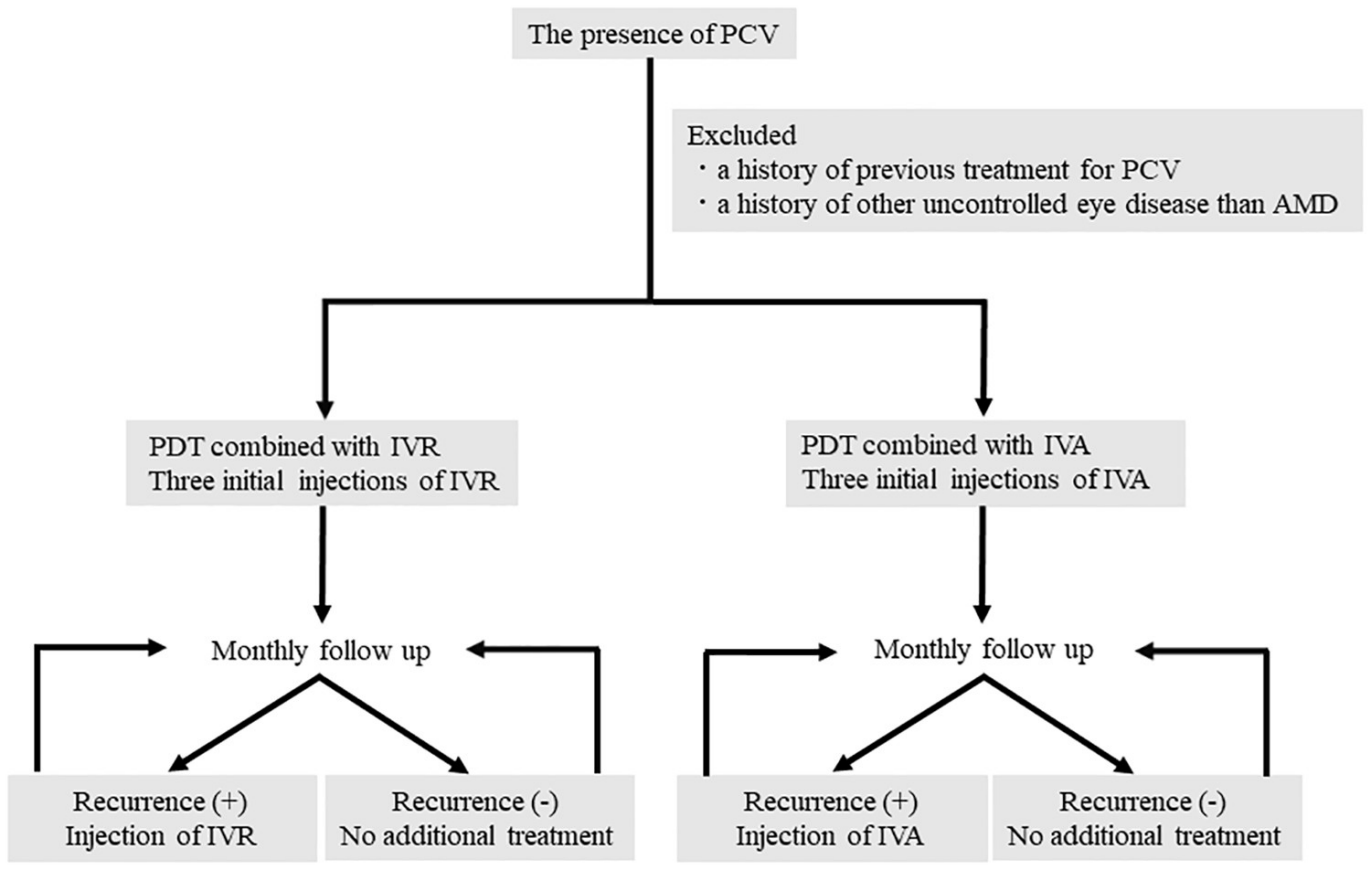
Flow diagram of inclusion and exclusion criteria and treatment schedule.

The patients received PDT combined with either IVR (PDT/IVR group) or IVA (PDT/IVA group). The anti-VEGF agents were chosen according to time period. From April 2017 to December 2017, IVR was administrated and from January 2018 to September 2018 IVA was administrated. Photodynamic therapy with verteporfin (Visudyne, Novartis, Basel, Switzerland) was administered according to the TAP Study protocol [22] except for the determination of the great linear dimension (GLD). Verteporfin was administered over 10 minutes. 15 minutes after the intravenous infusion started, a 689-nm laser was applied for 83 seconds to deliver 50 J/cm2. The GLD was determined based on the ICGA findings instead of the FA findings. The branching vascular network and polypoidal lesions were included in the GLD. A particular anti-VEGF agent was randomly selected by other person who wasn’t involved in this study. Patients in both groups received three initial monthly injections of anti-VEGF as the loading phase and PDT within 1 week after the initial injection. After the loading phase, all patients received monthly follow-up examinations and injections of anti-VEGF by *pro re nata* (PRN) regimen. [14] IVR or IVA was repeated in cases of the persistence or recurrence of subretinal fluid (SRF), intraretinal edema, or the deterioration of pigment epithelial detachment on the SD-OCT images and/or clinically perceptible hemorrhages (Fig 1).

The primary outcome measure was the change in best-corrected visual acuity (BCVA). The secondary outcome measures were the changes in central macular thickness (CMT), central choroidal thickness (CCT) measured manually using the caliper function in the SD-OCT, the number of intravitreal injections during a maintenance phase, and the regression of polyps evaluated by ICGA after 1 year.

CMT was defined as the distance between the internal limiting membrane and the RPE at the fovea. CCT was defined as the thickness between the Bruch’s membrane and the inner surface of the choroidal-scleral junction at the fovea. The great linear dimension (GLD) was measured using digital simultaneous FA and ICGA with a confocal scanning laser ophthalmoscope.

The statistical analysis software used was Excel Toukei 2012 (Social Survey Research Information, Tokyo, Japan). The Wilcoxon signed-rank test was used to compare pre-treatment and post-treatment BCVA, CMT, and CCT in each group. Baseline characteristics were compared using the Mann–Whitney *U* test and Fisher’s exact probability test. The BCVA was converted to logMAR values for the statistical analysis. *P* value <0.05 was considered to denote statistical significance.

## Results

The patients’ clinical data prior to treatment are presented in Table 1. Of the 44 patients enrolled in this study, 23 were in the PDT/IVR group, and 21 were in the PDT/IVA group. There were 35 men and nine women. The mean age was 73.3 ± 0.99 years (mean ± standard error [SE]) (range: 55 to 87 years). There was no significant difference between the two groups in age, sex ratio, baseline logMAR BCVA, CMT, CCT, and GLD (Table 1).

**Table 1:** Patient characteristics of all study eyes at baseline.

	PDT/IVR group	PDT/IVA group	*P-*Value
**Number of eyes**	23	21	
**Number of patients**	23	21	
**Male/Female**	17/6	18/3	0.27
**Age, mean ± SE, year (range)**	72.2 ± 1.34 (55–82)	74.6 ± 1.45 (55–87)	0.34
**logMAR BCVA, mean ± SE (range)**	0.23 ± 0.04 (0–0.82)	0.27 ± 0.08 (-0.079–1.096)	0.68
**CMT, mean ± SE (*μ*m) (range)**	361 ± 26 (204–716)	439 ± 35 (173–817)	0.055
**CCT, mean ± SE (*μ*m) (range)**	287 ± 15 (120–427)	261 ± 20 (120–437)	0.41
**GLD, mean ± SE (*μ*m) (range)**	3413 ± 245 (1131–7438)	3374 ± 208 (1701–4607)	0.63

PDT = photodynamic therapy; IVR = intravitreal ranibizumab; IVA = intravitreal aflibercept;

SE = standard error; BCVA = best-corrected visual acuity; CMT = central macular thickness;

CCT = central choroidal thickness; GLD = great linear dimention.

*P* Value indicates the statistical significance of difference between the group.

**Fig 2:**
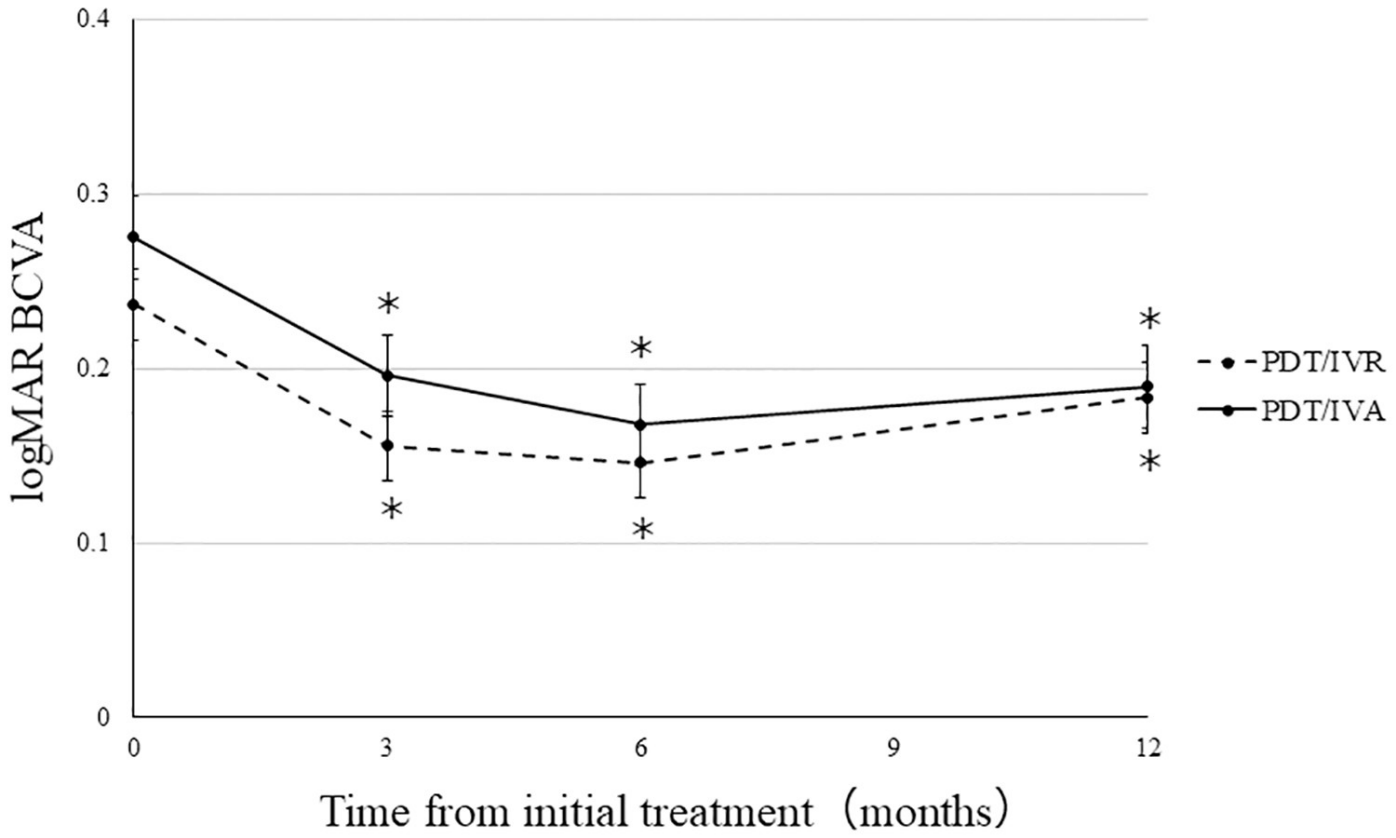
Changes in best-corrected visual acuity (BCVA) during the 12 month follow-up period. In both the PDT/IVR and PDT/IVA groups, the mean BCVA at 3, 6, and 12 months had improved significantly compared with the preoperative visual acuity (*P < 0.05).

In the PDT/IVR group, the mean logMAR BCVA significantly improved from 0.23 ± 0.04 prior to the initial treatment to 0.15 ± 0.05 at 3 months after the initial treatment (*P* = 0.014). The mean logMAR BCVA was maintained at 0.14 ± 0.06 (*P* = 0.010) after 6 months and 0.18 ± 0.08 (*P* = 0.022) after 1 year. In the PDT/IVA group, the mean logMAR BCVA significantly improved from 0.27 ± 0.08 prior to the initial treatment to 0.19 ± 0.07 at 3 months (*P* = 0.038) and was maintained at 0.16 ± 0.06 (*P* = 0.018) after 6 months and 0.18 ± 0.07 (*P* = 0.018) after 1 year (Fig 2). Increases in BCVA after 1 year did not differ between the PDT/IVR group (−0.05 ± 0.05) and the PDT/IVA group (−0.08 ± 0.03) (*P* = 0.58, Fig 5[A]).

**Fig 3:**
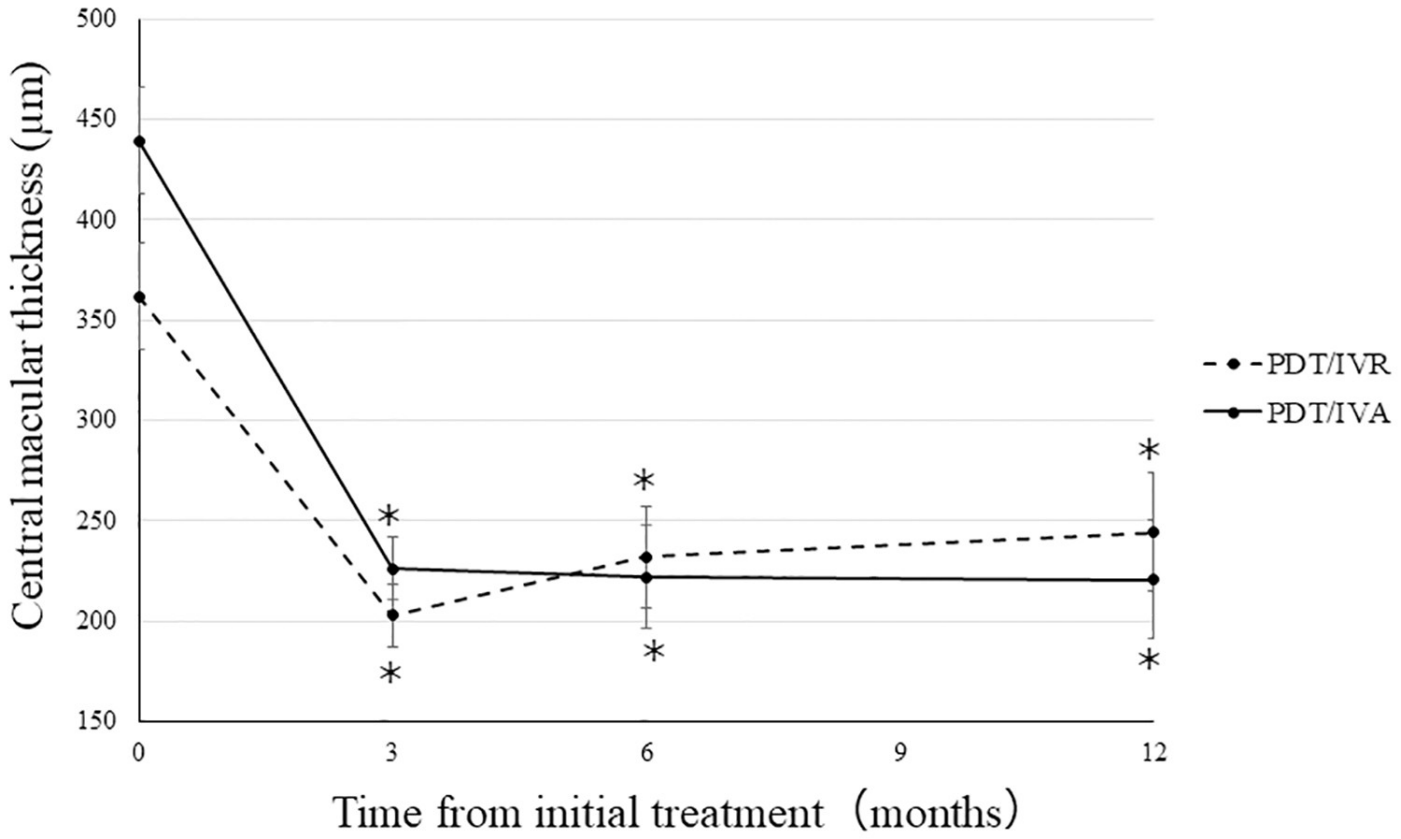
Changes in the central macular thickness (CMT) during the 12 month follow-up period. The mean CMT at 3, 6, and 12 months decreased significantly compared with those at baseline in the PDT/IVR and PDT/IVA groups (*P < 0.001).

In the PDT/IVR group, CMT significantly decreased from 361 ± 26 μm prior to the initial treatment to 202 ± 15 μm at 3 months (*P* < 0.001) and was maintained at 231 ± 25 μm (*P* < 0.001) after 6 months and 244 ± 29 μm (*P* < 0.001) after 1 year. In the PDT/IVA group, CMT significantly decreased from 439 ± 35 μm prior to the initial treatment to 226 ± 24 μm at 3 months (*P* < 0.001) and was maintained at 222 ± 17 μm (*P* < 0.001) after 6 months and 220 ± 15 μm (*P* < 0.001) after 1 year (Fig 3). The changes in CMT after 1 year did not differ between the PDT/IVR group (−117 ± 22) and the PDT/IVA group (−218 ± 35) (*P* = 0.06, Fig 5[B]).

**Fig 4:**
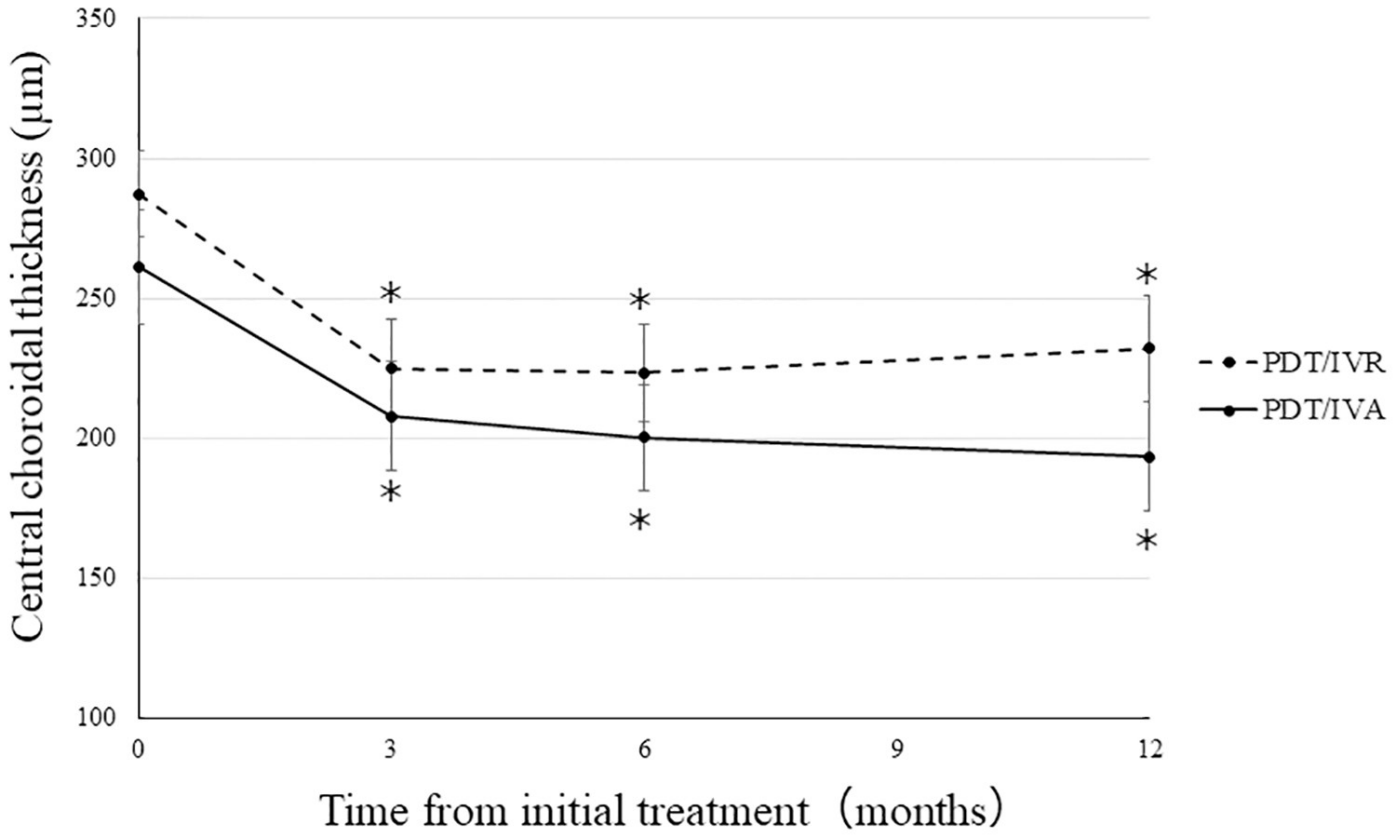
Changes in the central choroidal thickness (CCT) during the 12 month follow-up period. The mean CCT at 3, 6, and 12 months decreased significantly compared with those at baseline in the PDT/IVR and PDT/IVA groups (*P < 0.001).

**Fig 5:**
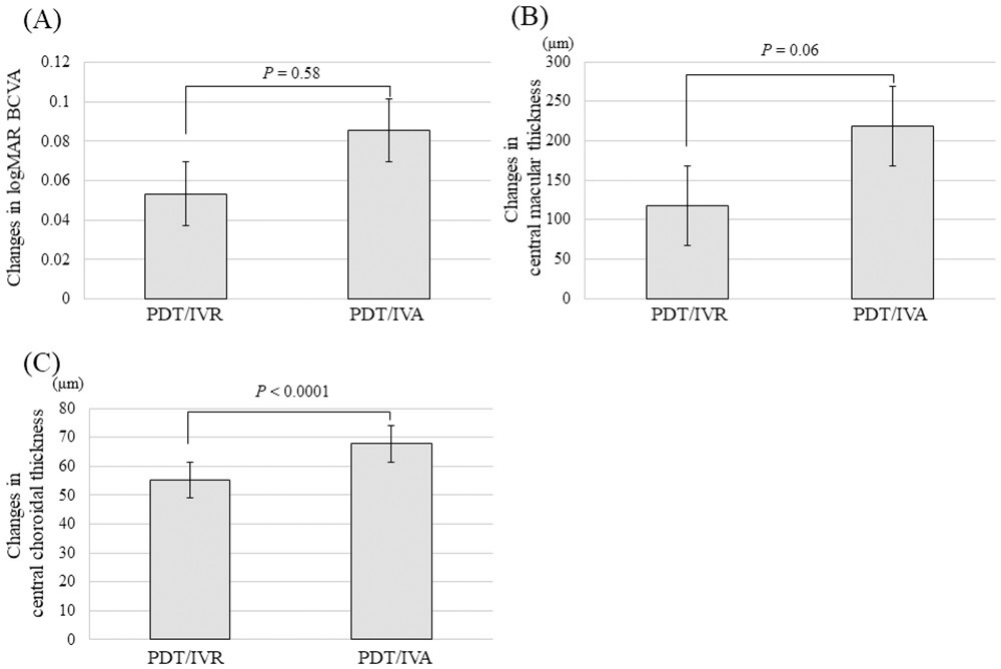
(A) Changes in logMAR BCVA after 1 year. The rate of BCVA increase during 1 year did not differ between the PDT/IVR (−0.05 ± 0.05) and PDT/IVA groups (−0.08 ± 0.03) (*P* = 0.58). (B) Changes in CMT after 1 year. The changes in CMT during 1 year did not differ between the PDT/IVR (−117 ± 22) and PDT/IVA groups (−218 ± 35) (*P* = 0.06). (C) Changes in CCT after 1 year. The changes in CCT during 1 year were significantly larger in the PDT/IVA group (−67 ± 12) than in the PDT/IVR group (−55 ± 9) (*P* < 0.001).

In the PDT/IVR group, CCT significantly decreased from 287 ± 15 μm prior to the initial treatment to 225 ± 17 μm at 3 months (*P* < 0.001) and was maintained at 223 ± 17 μm (*P* < 0.001) after 6 months and 232 ± 18 μm (*P* < 0.001) after 1 year. In the PDT/IVA group, CCT significantly decreased from 261 ± 20 μm prior to the initial treatment to 208 ± 19 μm at 3 months (*P* < 0.001) and was maintained at 200 ± 18 μm (*P* < 0.001) after 6 months and 193 ± 19 μm (*P* < 0.001) after 1 year (Fig 4). The changes in CCT after 1 year were significantly larger in the PDT/IVA group (−67 ± 12) than in the PDT/IVR group (−55 ± 9) (*P* < 0.001, Fig 5[C]).

During the 1-year study period, the mean number of additional injections administered was 0.78 ± 0.21 in the PDT/IVR group and 0.57 ± 0.21 in the PDT/IVA group. There was no significant difference between the two groups (*P* = 0.45).

The proportion of the patients who showed complete polyp regression after 1 year using ICGA was 78.2% in the PDT/IVR group and 76.9% in the PDT/IVA group. There was no significant difference between the two groups (*P* = 0.57).

**Fig 6:**
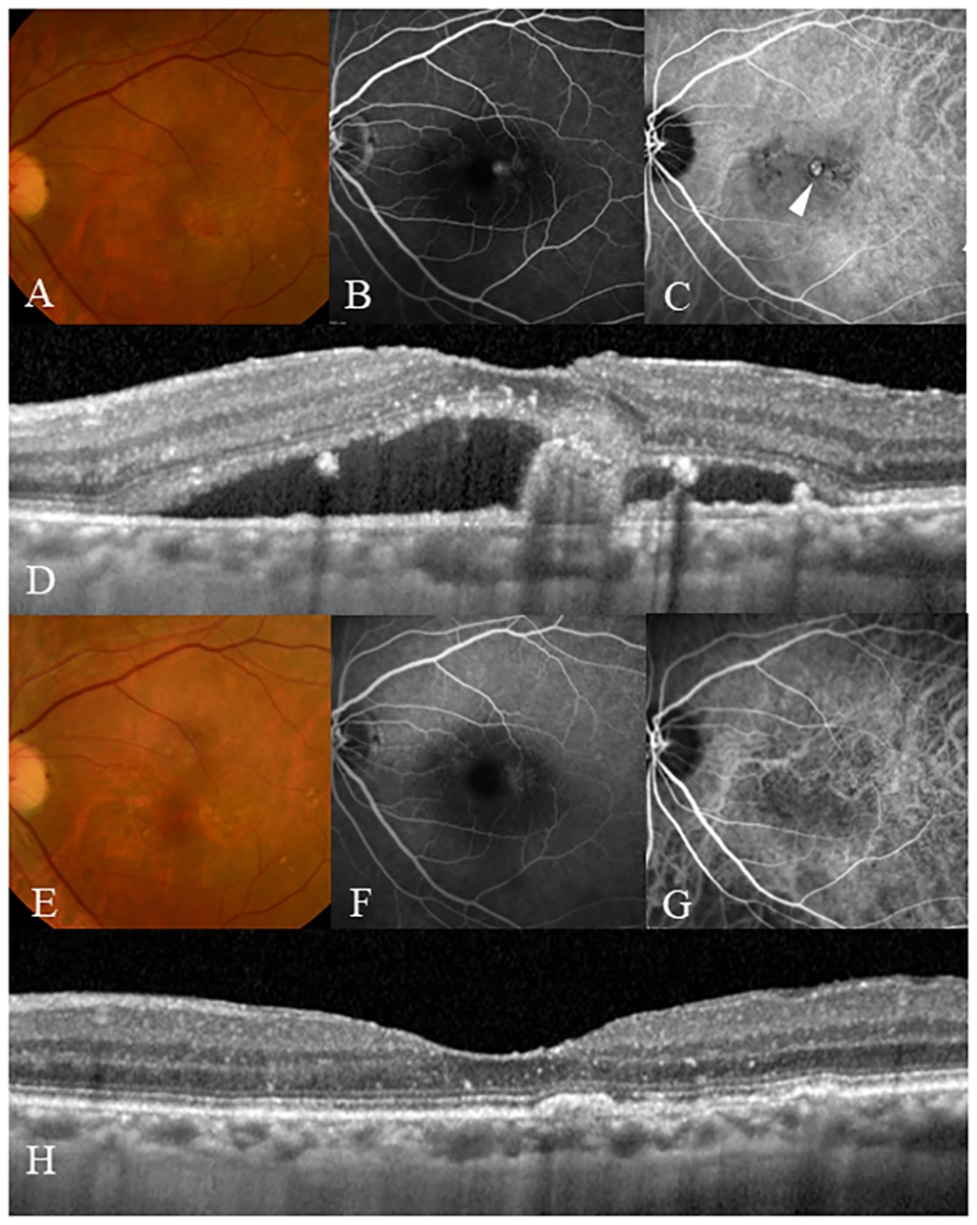
The case of a 79-year-old man who presented with reduced visual acuity in his left eye. (A) A color fundus photograph shows the reddish-orange polypoidal lesion, including his fovea, submacular hemorrhage, and a large area of subretinal fluid (SRF). (B) Early-phase fluorescein angiography (FA) revealed an occult leakage. (C) Early-phase indocyanine green angiography (ICGA) exhibits a polypoidal lesion (arrowhead) and an abnormal vascular network. (D) Optical coherence tomography (OCT) at the baseline shows SRF with a polypoid lesion. The visual acuity was 20/25 in the left eye, and the patient was diagnosed with PCV. The patient received PDT/IVA treatment during the loading phase, and during the maintenance phase, the exudative change did not recur. Twelve months after the first injection, the patient’s visual acuity improved to 20/16. (E) A color fundus photograph shows no reddish-orange lesion or hemorrhage at the macula. (F) Early-phase FA shows staining with no leakage. (G) Early-phase ICGA exhibits complete polyp regression, although an abnormal vascular network remained. (H) OCT shows no polypoidal lesion or SRF.

The exudative change did not recur in 13 eyes in the PDT/IVR group (56.5%) and 14 eyes in the PDT/IVA group (66.6%) during the maintenance phase. No incidence of serious ocular adverse effects, such as endophthalmitis, rhegmatogenous retinal detachment, or any nonocular adverse events, such as cerebral infarction, myocardial infarction, occurred. Fig 6 shows a representative case.

## Discussion

We found that BCVA in both groups was significantly improved, with few additional injections during the maintenance phase in patients with PCV. Moreover, CMT and CCT were significantly decreased after 1 year in both groups. There were no significant differences in the changes in BCVA, CMT, the number of additional injections, or regression rate of the polyps between the two groups; however, the changes in CCT in the PDT/IVA group were significantly larger than those in PDT/IVR group. There were few previous reports comparing PDT/IVR and PDT/IVA in patients with PCV.

Kikushima et al. reported that the PDT/IVA group demonstrated significantly greater visual improvement compared with the PDT/IVR group after 2 years. [21] However, their method differed from that of this study. They did not administer two additional injections in the loading phase and they basically followed every 3 months. Conversely, in our study, we administered three consecutive monthly injections in the loading phase and followed up according to PRN protocols. Much of the treatment in the loading phase and the strict follow-up in our study may result in no differences between how the two anti-VEGF agents improved BCVA.

In this study, after 1 year, CMT and CCT were significantly decreased in both groups. However, the changes in CCT in the PDT/IVA group were significantly larger than those in the PDT/IVR group, which is consistent with the results of previous studies. Maruko et al. reported that subfoveal choroidal thickness in patients with PCV decreased 1 month after patients received PDT and remained thin until 6 months after the initial treatment. [23] Kim et al. reported that there was a larger decrease in choroidal thickness in eyes treated with aflibercept compared with eyes treated with ranibizumab, and this difference was more remarkable in patients with PCV than those with other subtypes of AMD. [24] Even when patients are treated with a combination of PDT and anti-VEGF agents, CCT could become thinner by using aflibercept rather than ranibizumab. However, it was reported that a thin choroid might be a risk factor for the long-term development of chorioretinal atrophy (CRA) in patients with PCV. Choi et al. studied 88 patients with PCV who received anti-VEGF injections either with or without PDT. They reported that faster CRA growth was significantly related to a thin choroid, whereas PDT history was not significantly correlated with the development of CRA. [25] Thinning of the choroid could influence CRA in the longer term and CRA might be related BCVA. Therefore, it should be more cautious about CCT. Excessive choroidal thinning might further promote CRA, and it is necessary to consider whether it may affect the decrease of visual acuity.

In this study, the mean number of injections patients received over 1 year, including during the loading phase, was 3.78 in the PDT/IVR group and 3.57 in the PDT/IVA group. We previously reported the outcomes of a PRN regimen with IVA for treating PCV and reported that the mean number of IVA injections was 5.0 during 1 year. [14] Hikichi et al. reported the 1-year results of a PRN regimen combined with IVR to treat PCV and found that the mean number of IVR injections was 4.2. [8] Compared with IVA or IVR monotherapy, combination therapy in the form of a PRN regimen required fewer anti-VEGF injections. Furthermore, in the EVEREST Ⅱ study, which was a multicenter randomized clinical trial, compared with IVR monotherapy, treating PCV with IVR combined with PDT resulted in a greater increase in visual acuity with fewer numbers of IVR. [15] PDT combination therapy could reduce the number of anti-VEGF drug injections and lessen the treatment burden for both patients and doctors.

Importantly, it may be insufficient to evaluate the effect of treatment by increases in vision.; polyp regression must also be considered. In this study, complete polyp regression rates at 12 months did not significantly differ between the PDT/IVR group (78.2%) and the PDT/IVA group (76.9%). In the EVEREST Ⅱ study, the rates at 12 months were 69.7% in the patients who received PDT/IVR therapy and 33.8% in patients who received IVR monotherapy. In our previous study, when IVA monotherapy was used to treat PCV, the complete polyp regression rate was 48.0% with a bimonthly fixed regimen and 52.9% with a PRN regimen. [14] The polyp regression rate of patients who received combination therapy with either IVR and IVA was higher than either IVR or IVA monotherapy, which may have resulted in the fewer additional injections in the maintenance phase.

This study has some limitations. A major limitation is the retrospective design. The second limitation is the small sample size. Although there was no statistically significant difference in the baseline characteristics between the two treatment groups, it is necessary to perform a large-scale randomized study to confirm the current results. Furthermore, the long-term outcomes are unidentified. The results of this study must be evaluated with a longer follow-up period.

## Conclusion

PDT combined with either IVR or IVA effectively improved the vision and exudative changes in patients with PCV, over a 1-year follow-up. Although the PDT/IVA group showed a larger decrease in CCT than the PDT/IVR group, there was no significant difference in the visual improvement, the change in macular thickness or the number of additional injections between the two treatment groups after 1 year.
